# Outdoor Measurement and Modeling of Perovskite Module Temperatures

**DOI:** 10.1002/gch2.201800008

**Published:** 2018-05-28

**Authors:** Robert Gehlhaar, Tamara Merckx, Weiming Qiu, Tom Aernouts

**Affiliations:** ^1^ imec—Partner in Solliance Kapeldreef 75 ,3001 Leuven Belgium

**Keywords:** outdoor, perovskite, solar modules, temperature

## Abstract

Photovoltaic cells and modules are exposed to partially rapid changing environmental parameters that influence the device temperature. The evolution of the device temperature of a perovskite module of 225 cm^2^ area is presented during a period of 25 days under central European conditions. The temperature of the glass–glass packaged perovskite solar module is directly measured at the back contact by a thermocouple. The device is exposed to ambient temperatures from 3 to 34 °C up to solar irradiation levels exceeding 1300 W m^−2^. The highest recorded module temperature is 61 °C under constant high irradiation levels. Under strong fluctuations of the global solar irradiance, temperature gradients of more than 3 K min^−1^ with total changes of more than 20 K are measured. Based on the experimental data, a dynamic iterative model is developed for the module temperature evolution in dependence on ambient temperature and solar irradiation. Furthermore, specific thermal device properties that enable an extrapolation of the module response beyond the measured parameter space can be determined. With this set of parameters, it can be predicted that the temperature of the perovskite layer in thin‐film photovoltaic devices is exceeding 70 °C under realistic outdoor conditions. Additionally, perovskite module temperatures can be calculated in final applications.

## 1 Introduction

With record efficiencies of perovskite solar cells surpassing 23% of this thin‐film technology is approaching the efficiencies of the best devices of the established photovoltaic (PV) industries that use Si, copper indium gallium selenide (CIGS), or CdTe as photoactive materials.^1^ Potentially low‐cost fabrication processes and a short energy payback time can bring perovskite solar cells in a competitive market position.^2^, ^3^ The projected energy payback times are directly linked to device efficiency and stability. Especially, the device stability has been shown to be temperature dependent for perovskite solar cell architectures.^4^, ^5^, ^6^ For some device architectures the efficiency is far less temperature dependent due to voltage temperature coefficients in the range of 200–3000 ppm K^−1^,^7^, ^8^, ^9^ whereas higher temperature coefficients are also reported reducing the device performance at 85 °C remarkably.^10^ Strong temperature‐dependent performance changes have also been attributed to changes in the perovskite unit cell distortion, which influence the device hysteresis.^11^ Other temperature‐dependent hysteresis investigations indicate that changes in interface properties and transport layer mobilities alter the perovskite solar cell performance.^12^

In order to optimize future generations of commercial perovskite solar cells, precise temperature measurements of the layer stack under operation allow for a more precise analysis and modeling of the operational perovskite solar cell performances.

In the present work, we expose a setup with a perovskite module to outdoor conditions while tracking the module temperature directly measured at the back surface of the thin‐film stack.

## 2 Results

### 2.1 Thermal Measurement Configuration

**Figure 1:**
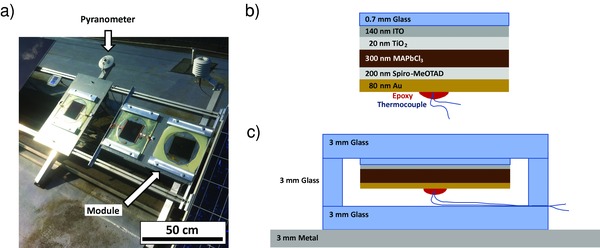
Photograph of a) the outdoor setup and b) illustrative cross sections of the planar perovskite module stack with attached thermocouple, and c) of the packaged and mounted device in superstrate configuration. Light is incident from the top in panels (b) and (c). The dimensions are not to scale.

The perovskite module temperature is measured on a 225 cm^2^ sample device that is mounted on a 2400 cm^2^ unpolished metal plate of stainless steel (**Figure** **1**a). The perovskite stack in planar configuration is identical to functional solar cells' stack as in electrically working solar modules (Figure 1b).^13^ In the glass–glass package (Figure 1c), a thermocouple is attached by an epoxy glue to the gold back electrode for a precise recording of the perovskite layer temperature. In this configuration, photon energy is absorbed with different efficiency by the perovskite module, the glass encapsulation, and the metal plate due to different absorption coefficients α. The highest absorption is expected for the perovskite device with an estimated $\alpha_{\mathrm{Per}}\approx \;0.9$ whereas for metal the estimated value is $\alpha_{\mathrm{steel}}\approx 0.3-0.4$.^14^ The thermal emissivity ε of glass and steel is close to unity.^15^

### 2.2 Measurements

**Figure 2:**
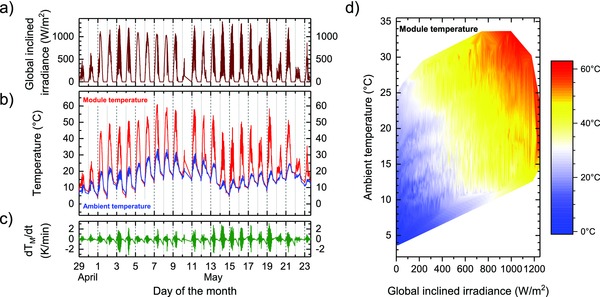
Measured outdoor data recorded during 25 days in 2016 of the perovskite module: a) pyranometer global inclined irradiance, b) module temperature and ambient temperature, c) temporal gradient of module temperature, and d) dependence of module temperature on ambient temperature and irradiance.

The measurement data cover a wide range of solar irradiance with particularly high values due to the recording period and sample inclination. With a south orientation and an inclination of 35°, the solar angle was nearly normal to the PV module at local noon. At times without cloud coverage, global inclined irradiance values above 1300 W m^−2^ have been measured (**Figure** **2**a). The maximum value of 1390 W m^−2^ is higher than it can be expected from horizontal irradiance data with maximum values of 1000–1050 W m^−2^ for the location of the setup. Due to the inclined position of the pyranometer, the albedo of the surrounding reflective metal roof top covering has increased the inclined global irradiance above its horizontal value. The highest value is confirmed by the measured module temperature which coincides with a high value of 58 °C despite the ambient temperature of only 18 °C (Figure 2b). During recording, the ambient temperature was varying from temperatures as low as 3 °C at night time up to maximum temperatures of 34 °C. For the perovskite module, maximum temperatures of 61 °C have been detected at a time of high ambient temperature (33 °C) and a longer period of high solar irradiance (1050 W m^−2^).

Figure 2c depicts the temporal gradient of the module temperature during the 25 days recording period. At times of highly varying solar irradiance due to rapidly changing cloud coverage of the sun, the temperature of the module changes with rates of up to 3.1 K min^−1^ in either direction. Exemplary, during such an event, the module temperature increased 25 K within 20 min.

### 2.3 Modeling

In order to simulate the dynamic module temperature evolution, we apply a model that uses the measured variables of the irradiant power *P*_irr_ and ambient temperature *T*_amb_ (Equation (1)). Since the temperature of the module is measured at only one single spot on the module, the complete assembly of mounting, metal plate, and glass–glass packaged module is considered as a homogeneous device with the absorption coefficient α, the emissivity ε, the specific thermal capacity *C*, and the front surface area *A*. At the beginning of every daily measurement interval at 05:00, the module temperature is assumed to be equal to the ambient temperature. For each measurement with a time step, Δ*t*, of normally 60 s, the energy balance is calculated by assuming that the thermal energy of the module assembly with the temperature *T*_M1_ and the specific thermal capacity *C* at the beginning of the interval are equal to the thermal energy of the module with the temperature *T*_M2_ after Δ*t*. In our iterative model, *T*_M2_ of the previous interval is identical to *T*_M1_ of the following interval. The energy of the module is increased by the incident power that is absorbed with a fraction equivalent to the absorption coefficient. In case the module is not in thermal equilibrium with its environment, we include the radiative thermal energy exchange by considering the emissivity of the module assembly and the Stefan–Boltzmann constant σ. For this term, we assume, for the simplicity of the equation, that the temperature of the module remains constant during Δ*t* with *T*_M1_.

(1) $$\begin{array}{l} T_{M2}(t)=\;T_{M1}(t)+\frac{\alpha A}{C}P_{\mathrm{irr}}(t)\Delta t\\ \;\;\;\;\;\;\;\;\;\;\;-\frac{2\epsilon A}{C}\sigma [T_{M1}^{4}(t)-T_{\mathrm{amb}}^{4}(t)]\Delta t \end{array}$$

**Figure 3:**
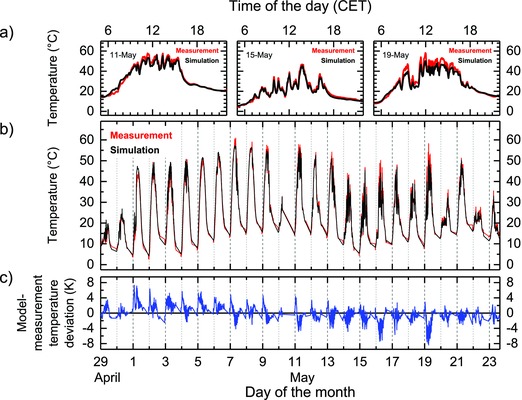
Comparison of measured data and simulation of temporal module temperature evolution of a) three selected days, b) the complete 25 day record, and c) the model–measurement deviation.

The two terms *αA*/*C* and *εA*/*C* are the specific temperature coefficients of the test assembly for radiative absorption and emission, respectively. Their values are obtained by fitting the complete measured set of nearly 25 000 individual data points. The average deviation for each data point between model and measurement is less than 1.5 K for the absorption temperature coefficient value of 4.52 × 10^−5^ K m^2^ J^−1^ and a temperature coefficient for the emission of 1.25 × 10^−4^ K m^2^ J^−1^. Comparing the model with the experimental data for three separate days and the full data set (**Figure** **3**a,b), we can demonstrate the simulation accuracy in tracing the particularly fast temporal changes of the perovskite module temperature. There is a day‐to‐day variation visible in the deviation of the model compared to the measurement (Figure 3c). On May 19, the day with the highest absolute deviations of up to 8 K, the model underestimates the module temperature despite following the temporal trend accurately. These high deviations that are also observed on other days are a consequence of the model simplification by not including conductive thermal effects due to a variation of atmospheric conditions. The reason for this approach is the unavailability of wind data for the test site. The non‐negligible wind influence is indicated by online wind data recorded in 18 km distance, which show remarkably different wind conditions for May 16 and May 19 from the other days with lower model deviations.^16^ A number of other thermal models have been applied in the data fitting with less accuracy in the result. The implementation of a term that represents a thermal conduction proportional to *A*(*T*_M1_ −*T*_amb_) showed no increased model accuracy over the approach by Equation (1).

When eliminating the time dependencies in Equation (1), the steady‐state module temperature *T*_M_ is derived.

(2) $$T_{M}={\left[\frac{\alpha P_{\mathrm{irr}}}{2\epsilon \sigma }+T_{\mathrm{amb}}^{4}\right]}^{\frac{1}{4}}$$

**Figure 4:**
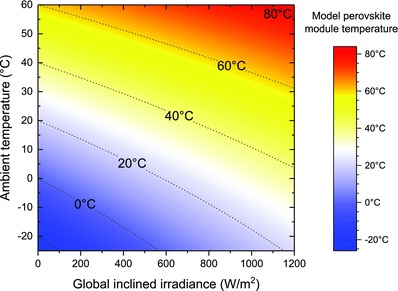
Simulation of the steady‐state module temperature in dependence on ambient temperature and global inclined irradiance. The calculations are based on device parameters derived from the fitting of the transient temperature data.

The influence of the irradiance power *P*_irr_ depends on the ratio of α and ε which is 0.36 in our sample setup. With this number, it is possible to calculate the module temperature for various irradiances and ambient temperatures (**Figure** **4**). Exemplary, we can derive a *T*_M_ of 59 °C for an ambient temperature of 35 °C and a constant solar irradiance of 1000 W m^−2^.

## 3 Discussion

In the test setup, a large area of the light‐absorbing area is consisting of highly reflective steel resulting in an overall low α, whereas the emissivity of steel and glass is in the range of 0.9–1.^15^ In the case of a perovskite module covering the full area, the absorption coefficient of the device can increase to 0.8. In such a more realistic solar cell configuration, the expected temperature for the same parameter combination of *T*_amb_ and *P*_irr_ can exceed 90 °C when α/ε ≈ 1.

The presented data set and model demonstrate the possibility of relevant device property extraction from a large data set of a small set of measured parameters. By recording ambient and module temperatures in combination with the global irradiance on the test device, the radiative parameters of absorption and emission in combination with the thermal capacity can be extracted with good accuracy. This set of device parameters allows for the precise estimation of the perovskite module temperature under steady‐state and temporal changing conditions. Based on the results of this work, perovskite modules can be expected to reach perovskite layer temperatures above 90 °C under extreme ambient temperatures (>40 °C) and high solar irradiance. For future developments of perovskite photovoltaic modules, the device stability at high efficiency levels is essential for applications to gain the economically necessary market share. In the past, some perovskites as well as hole and electron transport materials showed performance decays at temperatures below the here‐extrapolated temperatures.^4^, ^6^, ^11^, ^17^ New material and stack developments have to consider the layer temperatures and their potential fluctuation. During this development, it is of high relevance to test the solar cell performance and the long‐term stability at elevated device temperatures, especially if free‐standing devices as in the test setup are the target application. The ISOS test protocols provide recommended guidelines for this purpose.^18^ In building integration, lower module temperatures can be expected since the carrying architecture provides a large thermal capacity and thus reduces the temperature maximum and additionally decreases the absolute values of the temperature gradients below the values observed in this work. With current stack and stability developments we can be optimistic that perovskite solar modules can also pass the barrier of high operation layer temperatures.^19^

## 4 Experimental Section

*Module Preparation and Assembly*: Float glass substrates of 0.7 mm thickness coated with 140 nm ITO were cleaned subsequently with Extran, acetone, and isopropanol in ultrasonic baths. Afterward, 20 nm TiO_2_ was vacuum‐deposited by reactive electron beam evaporation under high vacuum. A 300 nm photoactive layer of methyl‐ammonium lead chloride followed by a 200 nm hole transporting layer of 2,2′,7,7′‐Tetrakis[*N*,*N*‐di(4‐methoxyphenyl)amino]‐9,9′‐spirobifluorene (Spiro‐MeOTAD) were deposited by blade coating with a film applicator Multicator 411 on an Erichsen Coatmaster 510 with coating speeds of 2.5 and 20 mm s^−1^, respectively. The planar perovskite solar device was finished by a vacuum‐deposited film of 80 nm gold as back electrode. A thermocouple was attached to the gold back electrode with a UV‐cured epoxy adhesive in the geometric center of the module. The encapsulation consisted of three layers of 3 mm thick glass with lateral dimensions of 30 × 30 cm^2^ that were connected by a UV‐cured epoxy glue with low water vapor and oxygen transmission rates. The middle glass plate had a central part removed in order to mount the perovskite module. The perovskite device was attached with the glass carrier surface facing light incidence to the top packaging glass. For optimal optical and mechanical connection, a UV‐cured solvent‐free adhesive was used. The electrical wires of the thermocouple were fed through the packaging within the epoxy glue connection. The glass–glass packaged module was mounted with two metal angular rails to a 3 mm thick metal plate from aluminum alloy with 40 × 60 cm^2^ dimension. This plate was fixed to a rail system of an outdoor test bench.

*Outdoor Test Bench*: The outdoor measurements were performed by the ENGIE Laborelec Solar lab at 50.76°N, 4.35°E. The perovskite module was mounted on a roof‐top outdoor test bench oriented South with 35° inclination. Beside the perovskite module, a pyranometer was mounted with the same inclination for the recording of the global inclined irradiance. The temperatures and irradiance data were recorded with timesteps of 1 min from 05:00 to 22:00 for a period of 25 days from April 29, 2016 to May 23, 2016. Times and dates were given in Central European Time.

## Conflict of Interest

The authors declare no conflict of interest.
